# Possible bronchospasm relief following flumazenil administration in a remimazolam-induced Grade III hypersensitivity reaction: a case report

**DOI:** 10.1186/s13256-026-06187-5

**Published:** 2026-06-09

**Authors:** Yunfei Shu, Wen Sun, Deyou Sun

**Affiliations:** Mianyang Key Laboratory of Anesthesia and Neuroregulation, Department of Anesthesiology, Mianyang Central Hospital, Mianyang, 621000 China

**Keywords:** Remimazolam, Anaphylaxis, Bronchospasm, Flumazenil, Case report, Procedural sedation

## Abstract

**Background:**

Remimazolam is an ultrashort-acting benzodiazepine for procedural sedation, with rare reports of anaphylaxis. Its clinical phenotypes and management strategies for refractory cases with suboptimal adrenaline response remain poorly defined. As remimazolam use increases globally, exploring effective interventions for its induced hypersensitivity reactions is clinically important. This report presents a rare remimazolam-induced Grade III hypersensitivity reaction dominated by bronchospasm, with flumazenil administration temporally associated with improvement of adrenaline-refractory symptoms, adding a hypothesis-generating observation to the existing literature.

**Case presentation:**

A 49-year-old Han female developed a Grade III hypersensitivity reaction after remimazolam administration for gastroscopy sedation, with no prior allergic history or underlying diseases. The main symptoms included severe persistent bronchospasm (initial and dominant manifestation), facial flushing, nausea, vomiting, abdominal pain, and hemodynamic instability (peripheral oxygen saturation 83%, blood pressure 75/53 mmHg, tachycardia). Discontinuation of remimazolam and stepwise adrenaline bolus administration failed to relieve bronchospasm effectively. Intravenous flumazenil administration, combined with an additional adrenaline dose, was temporally associated with the gradual resolution of all allergic symptoms and hemodynamic stabilization. The adrenaline dosing followed international perioperative anaphylaxis management consensus, with stepwise escalation based on the patient’s clinical response and American Society of Anesthesiologists physical status I.

**Conclusions:**

Remimazolam-induced hypersensitivity reactions can present as Grade III cases dominated by bronchospasm, requiring prompt clinical recognition and targeted intervention. The temporal association between flumazenil administration and relief of adrenaline-refractory bronchospasm warrants further investigation, but this observation is hypothesis-generating only; the independent therapeutic effect and mechanism of flumazenil remain unproven and require confirmation by more studies. For suspected remimazolam-induced anaphylaxis, serum tryptase measurement and convalescent allergen skin testing are recommended to confirm diagnosis and guide subsequent anesthetic selection. This single case report has inherent limitations, and clinical improvement may result from the combined effect of multiple interventions, so no definitive conclusion can be drawn regarding flumazenil's role.

## Introduction

Anaphylaxis is a potentially fatal systemic hypersensitivity reaction characterized by the rapid onset of symptoms affecting multiple organ systems and necessitates immediate therapeutic intervention [1]. Remimazolam is a novel ultrashort-acting benzodiazepine sedative-hypnotic agent approved in Japan in January 2020 and Europe in March 2021, indicated for procedural sedation and induction/maintenance of general anesthesia [2, 3]. The drug exerts sedative effects via positive allosteric modulation of the gamma-aminobutyric acid type A receptor (GABAₐ) receptor and is rapidly metabolized by tissue esterases to an inactive metabolite, endowing it with favorable properties of rapid onset, predictable recovery, and minimal cardiorespiratory depression [3].

Although perioperative anaphylaxis (POA) induced by remimazolam has been documented in several reported cases [1, 2], the full spectrum of its clinical characteristics and evidence-based management remain incompletely understood, and there is still a lack of unified treatment strategies for refractory cases with suboptimal response to adrenaline. In this report, we present a case of remimazolam-induced Grade III anaphylaxis in which the patient presented with severe bronchospasm, facial flushing, gastrointestinal symptoms, and hemodynamic instability. Notably, initial adrenaline administration provided only a partial response, and subsequent injection of flumazenil (concurrent with an additional adrenaline dose) was temporally associated with the gradual resolution of all allergic manifestations. These findings raise the hypothesis that flumazenil may have a potential role in the management of remimazolam-related anaphylaxis, particularly in cases refractory to conventional therapy and dominated by bronchospasm. However, its specific mechanism and clinical efficacy remain speculative, and no causal relationship has been established.

## Case summary

The patient was a 49-year-old female of Han ethnicity with no notable psychosocial factors—specifically, no history of mental disorders, stable family support, and no financial or social barriers to accessing medical care. She had no significant personal or familial medical history, including no documented atopy, asthma, or allergic disorders (for example, drug, food, or contact allergies) in herself or her family. Additionally, she denied any history of smoking, alcohol consumption, prior anesthesia exposure, or surgical procedures. The patient received no oral or injectable medications within 72 hours before the procedure, and no premedication (such as anticholinergics or antihistamines) was administered prior to gastroscopy. Upon admission, her vital signs were within normal ranges: body weight 50 kg, height 161 cm (body mass index 19.3 kg/m^2^), temperature 36.5 °C, blood pressure 124/81 mmHg, respiratory rate 18 breaths per minute, and heart rate 81 beats per minute. Physical examination revealed no remarkable abnormalities, and the patient was classified as American Society of Anesthesiologists (ASA) physical status I.

**Fig. 1:**
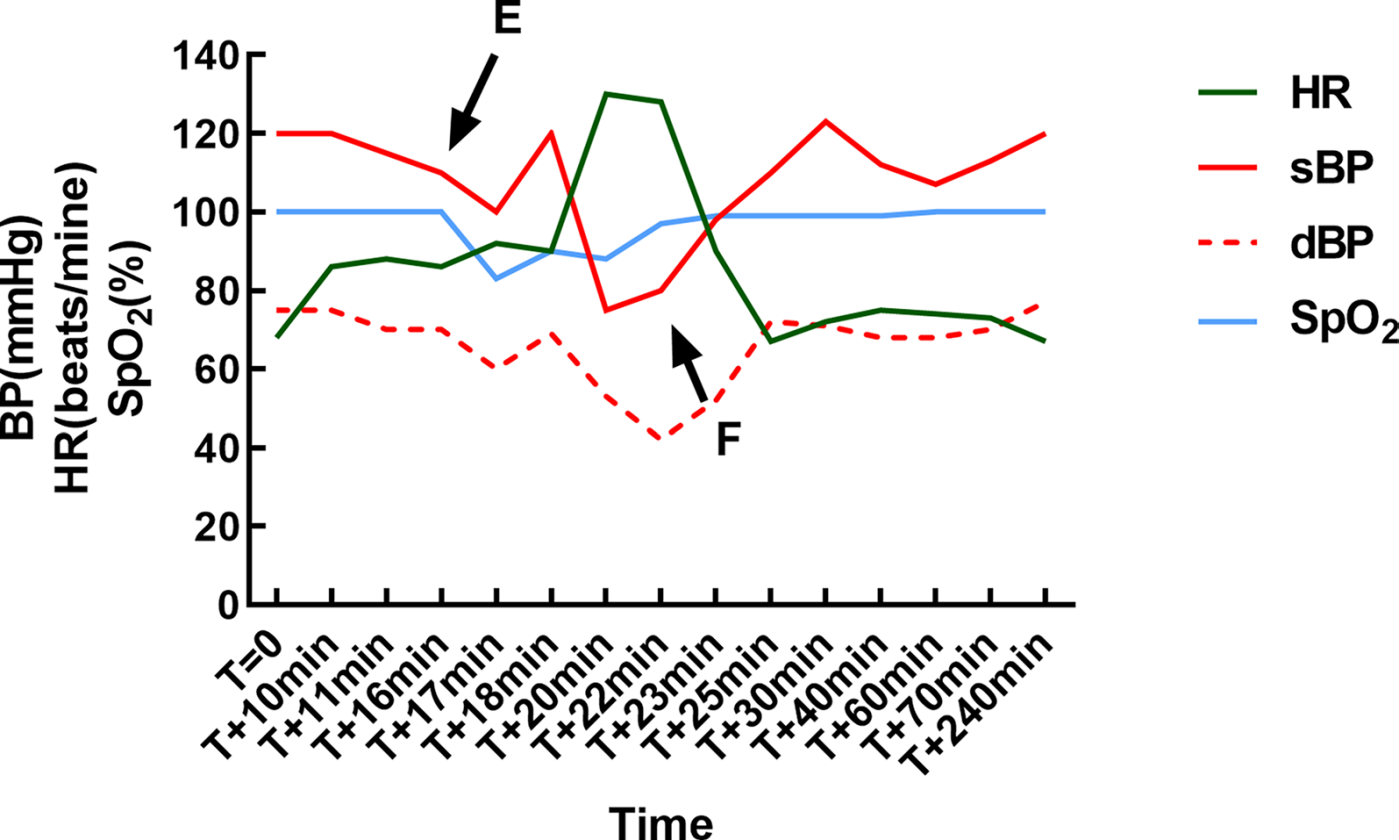
Dynamic changes in the patient's vital signs during the anaphylactic episode. BP: blood pressure; HR: heart rate; sBP: systolic blood pressure; dBP: diastolic blood pressure; E: the time point of epinephrine administration; F: the time point of flumazenil administration. The arrows indicate the times of initial epinephrine and flumazenil administration

Following standard preoperative fasting, the patient was transferred to the gastrointestinal endoscopy suite at T = 0. At T = + 10 minutes, remimazolam 5 mg and sufentanil 5 µg were administered intravenously as a stat dose; at T = + 11 minutes, a continuous intravenous infusion of remimazolam was initiated at 1 mg/kg/hour, and gastroscopy was subsequently commenced. A summary of the clinical workflow is provided in Table 1, and dynamic changes in vital signs are graphically represented in Fig. 1.

**Table 1:** Summary of medical consultation processes

Time	BP (mmHg)	HR (beats/minute)	SpO_2_ (%)	Symptoms and signs	Management
T = 0	120/75	68	100	Normal	Cardiac monitoring, O_2_ @ 5 L/minute per NC
T = + 10 minutes	120/75	86	100	Sedation, no adverse symptoms	Remimazolam (i.v. 5 mg st), sufentanil(i.v. 5 µg st)
T = + 11 minutes	115/70	88	100	Sedation, stable vital signs	Remimazolam (C.I. 1 mg/kg/hour st), gastroscopy
T = + 16 minutes	110/70	86	100	Facial erythema, suprasternal and supraclavicular retractions, wheezes	Discontinue remimazolam, epinephrine (i.v. 10 µg st)
T = + 17 minutes	100/60	92	83	Symptoms not relieved	Epinephrine (i.v. 10 µg st), aborted
T = + 18 minutes	120/69	90	90	Symptoms not relieved	Epinephrine (i.v. 20 µg st)
T = + 20 minutes	75/53	130	88	Symptoms not relieved	Epinephrine (i.v. 20 µg st)
T = + 22 minutes	80/42	128	97	Symptoms not relieved	Epinephrine (i.v. 20 µg st), flumazenil (i.v. 0.25 mg st)
T = + 23 minutes	98/52	90	99	Alert, dyspnea (C/O), resolving wheezes	No additional intervention
T = + 25 minutes	110/72	67	99	SOB improved (C/O),, minimal wheezes	No additional intervention
T = + 30 minutes	123/71	72	99	No wheezes, stable vital signs	Transfer to PACU
T = + 40 minutes	112/68	75	99	Dizziness, nausea, vomiting (C/O)	Tropisetron (i.v. 2 mg st)
T = + 1 hour	107/68	74	100	Abdominal pain	Atropine (i.v. 0.5 mg st)
T = + 1 hour 10 minutes	113/70	73	100	Abdominal pain improved	No additional intervention
T = + 4 hours	120/77	67	100	Normal	Discharge from PACU

Abbreviations: BP, blood pressure; HR, heart rate; SpO_2_, peripheral oxygen saturation; O_2_, oxygen; NC, nasal cannula; C/O, complains of; SOB, shortness of breath; PACU, post‑anesthesia care unit; i.v., intravenous; st, stat (immediate dose); C.I., continuous infusion

As detailed in Table 1, the patient developed acute anaphylactic symptoms between T = + 16 and T = + 22 minutes. During this period, a structured physical examination was performed, revealing the following findings: airway: patent, no laryngeal edema or stridor; lungs: bilateral diffuse expiratory wheezes, decreased air entry, suprasternal and supraclavicular retractions; skin: facial erythema, no urticaria or angioedema; cardiovascular: tachycardia (HR 128 beats/minute), hypotension (BP 80/42 mmHg), regular rhythm, no murmurs; abdomen: soft, mild tenderness on palpation, no rebound tenderness or guarding.

On post-event day 12, the patient underwent endoscopic submucosal dissection (ESD) under general anesthesia. Notably, neither remimazolam nor midazolam was administered during this procedure. Other anesthetic agents, including sufentanil, remifentanil, rocuronium, desflurane, ciprofol, and propofol, were administered within standard therapeutic dosage ranges, and no perioperative allergic manifestations were observed. All laboratory parameters, as detailed in Table 2, fell within normal reference ranges.

**Table 2:** Hematological parameters relevant to anaphylaxis evaluation

Blood routine test	T = −2 days (pre-procedure)	T = + 10 days (post-procedure)	Reference range
White blood cell count, × 10^9^/L	5	4.97	3.5–9.5
Neutrophil percent, %	65.3	75.3	40.0–75.0
Lymphocyte percent, %	25.4	19.4	20.0–50.0
Eosinophil percent, %	0.026	0.6	0.4–8.0
Eosinophil count, × 10^9^/L	0.12	0.03	0.02–0.52
Basophil percent, %	0.3	0.2	0.0–1.0
Basinophil count, × 10^9^/L	0.01	0.01	0.00–0.06
Hemoglobin assay, g/L	133	126	115–150
Platelet count, × 10^9^/L	133	140	100–300

The patient was followed up by telephone at 1 month and 3 months after discharge. She reported no recurrence of allergic symptoms, no respiratory discomfort during daily activities, and no adverse reactions related to the anesthetic agents used during ESD. She was referred to the hospital's allergy clinic for formal evaluation, but declined in-person visits and additional diagnostic tests (including serum tryptase reexamination and allergen skin testing) due to personal schedule constraints. A permanent alert was added to the patient's electronic medical record to avoid future exposure to remimazolam and midazolam; the future sedation plan was documented as preferring propofol-based regimens with pre-procedure risk assessment and close clinical monitoring.

## Discussion

We report a case of a 49-year-old female patient with gastric neuroendocrine tumors who developed Grade III anaphylaxis dominated by bronchospasm following the administration of a conventional dose of remimazolam for gastroscopy sedation. The patient's bronchospasm was relieved and hemodynamic parameters stabilized after flumazenil administration (concurrent with an additional adrenaline dose), and this clinical improvement was temporally associated with flumazenil use rather than indicating a definitive causal relationship. Although multiple cases of remimazolam-associated anaphylaxis have been documented in the literature [1, 6, 10], the majority occur during the induction of general anesthesia and exhibit a male predominance; hypotension, frequently accompanied by bradycardia and hypoxemia, is the most common documented clinical manifestation, with epinephrine as the mainstay of treatment [6]. For example, three patients developed severe circulatory collapse following endotracheal intubation under remimazolam anesthesia in the absence of cutaneous or respiratory symptoms. Hemodynamic stability was gradually restored in these individuals through fluid resuscitation, vasopressor support, epinephrine administration, and chest compressions [10]. In two additional cases, patients presented with rash, chest tightness, or dyspnea accompanied by significant hypotension within 2–3 minutes after intravenous induction, and epinephrine administered after conventional vasoactive agents proved ineffective, resulting in hemodynamic stabilization within minutes [10]. A previously published case under procedural sedation with remimazolam shared certain features with the present case; however, the patient developed severe laryngeal edema within one minute of drug injection, accompanied by audible stridor and suprasternal retraction. This was followed by extensive erythema involving the face, neck, and chest, along with labial and periorbital edema. Symptoms resolved following treatment with epinephrine, fluid resuscitation, and subsequent induction with propofol and vecuronium, followed by laryngeal mask insertion and mechanical ventilation, which allowed completion of the colonoscopy [1].

Perioperative anaphylaxis is a rare yet potentially life-threatening condition, with an estimated incidence of approximately 1 in 7000–10,000 anesthetic procedures [5] and a mortality rate ranging from 3% to 4.8% [6]. It manifests through a broad spectrum of clinical signs, ranging from mild cutaneous involvement to severe systemic reactions, and isolated cardiovascular collapse or cardiac arrest may serve as the initial indicator in some cases. Unexplained hypotension—with or without tachycardia—or hemodynamic resistance to vasopressors should raise clinical suspicion of anaphylaxis, particularly in patients taking β-blockers, who may present with bradycardia or an unchanged heart rate [4]. Prompt recognition and appropriate management by anesthesiologists are critical to ensuring favorable patient outcomes [7].

Remimazolam is a novel benzodiazepine sedative characterized by its rapid onset of action, short elimination half-life, and minimal depressant effects on respiratory and circulatory function [8]. Its sedative effects can be selectively antagonized by flumazenil [9], contributing to its growing use in sedation for various outpatient examinations and short-duration procedures [1]. However, owing to its relatively recent introduction into clinical practice, remimazolam-induced allergic reactions remain rarely reported. Here, we present a clinical case in which flumazenil was administered for remimazolam-induced Grade III anaphylaxis dominated by bronchospasm with suboptimal response to adrenaline, and the patient's bronchospasm was relieved and hemodynamic parameters were improved after flumazenil administration. It must be emphasized that the clinical improvement may be the combined effect of multiple factors, including repeated epinephrine administration, cessation of the remimazolam trigger, and the natural evolution of the anaphylactic reaction; the independent therapeutic effect of flumazenil remains unproven. Alternative explanations for symptom relief, such as delayed effect of epinephrine or spontaneous resolution, cannot be excluded.

### Confirmation of anaphylaxis

The diagnosis of anaphylaxis is principally based on the patient’s clinical history and evidence of multiorgan system involvement, with the modified Ring and Messmer scale serving as the gold standard for grading the severity of perioperative immediate hypersensitivity reactions [5]. Established risk factors for POA include male sex, emergency surgical procedures, a history of hypertension or other cardiovascular comorbidities, obesity, and beta-blocker use; antibiotics and neuromuscular blocking agents remain the most frequently implicated causal agents in such reactions. Serum tryptase is the most validated and widely used biomarker for corroborating a diagnosis of anaphylaxis, with acute levels ideally measured within 30 minutes of symptom onset and no later than 2 hours to maximize diagnostic accuracy [12]; baseline tryptase testing during the convalescent period further aids in distinguishing mast cell-mediated anaphylaxis from other hypersensitivity reactions.

Serum tryptase was not measured at the time of the anaphylactic reaction because the patient developed severe bronchospasm and profound hemodynamic instability within 5 minutes of remimazolam administration, and the clinical team prioritized immediate resuscitation to stabilize vital signs, which precluded timely blood sample collection. In addition, the patient declined formal allergen testing (including skin prick testing and intradermal testing for remimazolam) and baseline tryptase measurement during the follow-up period due to personal schedule constraints. Notably, the absence of serum tryptase measurement (a key biomarker for anaphylaxis) and formal allergy testing limits the definitive confirmation of mast cell-mediated anaphylaxis and the causal attribution to remimazolam. Although the clinical presentation and temporal association strongly suggest remimazolam as the culprit, the lack of objective laboratory evidence means the diagnosis remains based on clinical suspicion and exclusion rather than definitive proof. This limitation should be considered when interpreting the case, as non-immunologic mast cell activation or other non-anaphylactic hypersensitivity reactions cannot be completely ruled out.

Despite the lack of objective laboratory and confirmatory testing, the Grade III hypersensitivity reaction in this case was clinically diagnosed as remimazolam-induced anaphylaxis based on a clear temporal association and systematic exclusion of alternative causes. First, the acute onset of symptoms occurred immediately after remimazolam administration, with no other drugs or stimuli introduced in the peri-procedural period that could trigger a hypersensitivity reaction. Second, the patient presented with classic multiorgan involvement consistent with anaphylaxis: respiratory compromise (severe bronchospasm with hypoxemia [SpO_2_ 83%], suprasternal and supraclavicular retractions), cardiovascular instability (profound hypotension [80/42 mmHg] with tachycardia), cutaneous manifestations (facial erythema), and gastrointestinal symptoms (nausea, vomiting, abdominal tenderness). Third, alternative diagnoses were systematically ruled out: the absence of chest pain, arrhythmias, or abnormal cardiac biomarkers excluded a cardiac etiology; the lack of fever, leukocytosis, or purulent secretions ruled out acute respiratory infection; and the absence of a history of asthma or chronic obstructive pulmonary disease precluded an acute exacerbation of chronic airway disease. This constellation of multiorgan clinical features, including severe bronchospasm with hypoxemia (SpO₂ 83%) and profound hypotension (BP 75/53 and 80/42 mmHg with tachycardia), is highly consistent with a Grade III hypersensitivity reaction, as defined by the modified Ring and Messmer scale [5].

Following clinical diagnosis of anaphylaxis, identification of the causal agent was further supported by subsequent anesthetic exposure. During the index gastroscopy procedure, only remimazolam and sufentanil were administered prior to the onset of symptoms; in contrast, the patient underwent ESD on post-event day 12 with a multi-agent anesthetic regimen including sufentanil, remifentanil, rocuronium, desflurane, ciprofol, and propofol—with no remimazolam or midazolam administered—and no hypersensitivity or allergic manifestations were observed intraoperatively or postoperatively. This selective avoidance of benzodiazepines (specifically remimazolam) with uneventful exposure to all other previously administered agents provides compelling clinical evidence that remimazolam was the likely culprit of the acute hypersensitivity reaction.

### Analysis of treatment strategy and clinical improvement factors

From the clinical timeline, the patient received repeated intravenous epinephrine boluses before flumazenil administration, and the blood pressure showed a slight improvement trend before flumazenil was given. Flumazenil was administered concurrently with an additional 20 μg epinephrine, making it impossible to isolate the specific contribution of flumazenil from the cumulative effects of epinephrine and the natural self-limited recovery process. The role of flumazenil in this case is mainly reflected in the temporal association with rapid relief of bronchospasm, which is the most prominent and persistent symptom. This association should be viewed as hypothesis-generating for future research, not as evidence of a definitive therapeutic effect.

In this case, the epinephrine dosing adopted a stepwise small bolus escalation strategy based on the patient's clinical condition. The initial dose of 10 μg epinephrine was selected considering the patient's young age, ASA I physical status, and no underlying heart disease, to avoid adverse cardiovascular reactions caused by excessive epinephrine. The dose was escalated to 20 μg successively according to the patient's suboptimal clinical response, which is in line with the guideline recommendations for Grade III anaphylaxis [5]. However, it should be acknowledged that the administration of only bolus epinephrine without considering epinephrine infusion is a limitation of the treatment scheme in this case, which may be related to the urgent clinical scenario and the priority of relieving severe bronchospasm at that time.

The potential effect of flumazenil in relieving bronchospasm in this case is a clinical hypothesis that has not been confirmed by basic research, and there is currently no established mechanism by which flumazenil reverses mast cell-mediated anaphylaxis. We speculate that the potential effect of flumazenil may be related to its specific antagonistic effect on the GABAₐ receptor, which may indirectly regulate the bronchial smooth muscle tension and relieve bronchospasm, but this hypothesis lacks experimental evidence. Alternative explanations for the relief of bronchospasm include the delayed effect of epinephrine on bronchial smooth muscle and the reduction of airway inflammation with the natural evolution of the anaphylactic reaction. At present, the role of flumazenil in mast cell-mediated anaphylaxis has not been reported in relevant studies, and its specific mechanism and clinical application value need to be further studied by basic experiments and large-sample clinical observations.

Flumazenil is a specific benzodiazepine receptor antagonist that competitively inhibits the binding of benzodiazepines to GABAₐ receptor in the central nervous system, thereby blocking or reversing the pharmacological effects of benzodiazepines [9]. It is clinically indicated for the reversal of sedative and hypnotic effects, the amelioration of benzodiazepine-induced respiratory depression, and as a diagnostic aid in cases of unexplained coma resulting from benzodiazepine overdose [9]. In the present case, flumazenil was administered empirically as an antagonistic intervention, and the patient regained consciousness promptly with rapid relief of bronchospasm. This provides only a hypothesis-generating observation regarding the potential off-label use of flumazenil in remimazolam-induced anaphylaxis dominated by bronchospasm; no definitive conclusion can be drawn.

The management of suspected perioperative anaphylaxis involves three critical components: prompt recognition, judicious administration of epinephrine, and adequate fluid resuscitation [5]. Suboptimal or excessive treatment may lead to adverse outcomes, particularly in patients with significant cardiac or respiratory comorbidities, elevated ASA physical status, obesity, advanced age, or clonal mast cell disorders, as well as in those receiving β-adrenergic receptor blockers or angiotensin-converting enzyme (ACE) inhibitors. Currently, epinephrine remains the first-line treatment for anaphylaxis; however, its clinical application in conjunction with fluid resuscitation is often suboptimal in real-world practice [5, 11]. According to established guidelines, grade I anaphylaxis does not necessitate epinephrine administration. For Grade II anaphylaxis, the initial recommended epinephrine dose ranges from 10 to 20 μg, whereas for Grade III anaphylaxis, it is 50–100 μg. In patients exhibiting a suboptimal response to other vasopressors, bronchodilators, or both, an initial dose of 100 μg epinephrine is advised. If the clinical response remains inadequate after 2 minutes, the dose should be titrated upward to 50 μg for Grade II reactions and to 200 μg for Grade III reactions [5]. In cases of Grade IV anaphylaxis, the standard advanced cardiac life support (ACLS) protocol should be initiated immediately, including the intravenous bolus administration of epinephrine at 1 mg per dose [5]. Adequate fluid resuscitation is essential to mitigate decreased preload resulting from vasodilation and increased capillary permeability, with 0.5 L of crystalloid solution for Grade II and 1 L for Grade III anaphylaxis [5], and repeated boluses administered if the clinical response remains inadequate.

## Conclusions

The identification of drug-induced anaphylaxis remains clinically challenging, particularly in the perioperative setting where multiple agents are administered concurrently. Anaphylactic reactions to midazolam, etomidate, ketamine, and inhaled anesthetics are exceedingly uncommon, while remimazolam-induced anaphylaxis is an emerging clinical issue with the growing use of the drug. In the present case, remimazolam elicited a Grade III hypersensitivity reaction dominated by bronchospasm, accompanied by cutaneous, hemodynamic, and gastrointestinal manifestations. Notably, when epinephrine yielded a suboptimal response in relieving severe bronchospasm, the administration of flumazenil was temporally associated with the prompt resolution of bronchospasm and the gradual stabilization of hemodynamic parameters. However, the clinical improvement is likely the result of multiple synergistic factors, and the independent therapeutic effect of flumazenil cannot be confirmed; alternative explanations cannot be ruled out. This single case report provides only hypothesis-generating observations and should not be interpreted as evidence of efficacy.

This case provides a hypothesis-generating clinical observation for the management of remimazolam-induced Grade III anaphylaxis dominated by bronchospasm and reminds anesthesiologists to pay attention to the diverse clinical manifestations of remimazolam-induced anaphylaxis. In clinical practice, for patients with suspected remimazolam-induced anaphylaxis, it is recommended to measure serum tryptase as early as possible (within 2 hours of symptom onset) on the premise of ensuring hemodynamic stability; for patients with a clear clinical diagnosis of remimazolam anaphylaxis, skin testing can be performed during the convalescence period to further confirm the allergen and provide a reliable basis for the selection of anesthetic drugs for subsequent surgeries. A permanent alert should be added to the patient's electronic medical record to avoid future exposure to the culprit drug, and a personalized sedation plan should be formulated for subsequent procedures.

The main limitations of this study include the absence of objective confirmatory testing (serum tryptase measurement and allergen skin testing), which reduces the certainty of the diagnosis and definitive causal attribution; the over-reliance on bolus epinephrine in the treatment process without considering epinephrine infusion, which is a limitation of the clinical treatment scheme; and the lack of basic research to support the potential mechanism of flumazenil in relieving anaphylactic bronchospasm. In addition, as a single case report, the conclusions of this study cannot be generalized, and more large-sample clinical studies, case series, and basic mechanism research are needed to confirm the potential value of flumazenil in the treatment of such anaphylactic reactions. Future research should focus on exploring the underlying mechanism of flumazenil in relieving bronchospasm and conducting prospective clinical observations to verify its efficacy and safety.

## Data Availability

The datasets for this article are not publicly available owing to concerns regarding participant/patient anonymity. Requests to access the datasets should be directed to the corresponding authors.

## Abbreviations

POA: Perioperative anaphylaxis
ESD: Endoscopic submucosal dissection
BP: Blood pressure
HR: Heart rate
sBP: Systolic blood pressure
dBP: Diastolic blood pressure
GABAₐ: Gamma-aminobutyric acid type A receptor
ACE: Angiotensin-converting enzyme
ACLS: Advanced cardiac life support
ASA: American Society of Anesthesiologists
